# Structurally diverse biological nitrification inhibitors display distinct modes of inhibition in ammonia-oxidizing bacteria

**DOI:** 10.1093/femsec/fiag032

**Published:** 2026-03-25

**Authors:** Jasmeet Kaur-Bhambra, Purna K Khatri, Jawameer Hama, Cecile Gubry-Rangin, Kristian K Brandt

**Affiliations:** Department of Plant and Environmental Sciences, University of Copenhagen, Thorvaldsensvej 40, 1871 Frederiksberg C, Denmark; School of Biological Sciences, University of Aberdeen, Scotland, United Kingdom; Department of Agroecology, Aarhus University, Forsøgsvej 1, 4200, Slagelse, Denmark; Department of Agroecology, Aarhus University, Forsøgsvej 1, 4200, Slagelse, Denmark; School of Biological Sciences, University of Aberdeen, Scotland, United Kingdom; Department of Plant and Environmental Sciences, University of Copenhagen, Thorvaldsensvej 40, 1871 Frederiksberg C, Denmark

**Keywords:** ammonia-oxidizing bacteria, AOB, biological nitrification inhibition, Enzyme inhibition

## Abstract

Biological nitrification inhibitors (BNIs) are plant-derived compounds that suppress ammonia oxidation, representing a nature-based solution for reducing nitrogen losses and nitrous oxide emissions in agroecosystems. However, the modes of inhibition by which BNIs affect ammonia-oxidizing micro-organisms remain poorly understood. In this study, we examined the ammonia oxidation enzyme kinetics in two model species of ammonia-oxidizing bacteria (AOB), *Nitrosomonas europaea* and *Nitrosospira multiformis*, as affected by three structurally distinct BNIs: methyl 3-(4-hydroxyphenyl)propionate (MHPP), gallic acid (GA), and 6-methoxybenzoxazolinone (MBOA). GA acted as a potent, irreversible inhibitor, halting AOB activity within 1 h at 250 µM, with no recovery in cellular activity after inhibitor removal. In contrast, MHPP and MBOA exhibited a reversible mode of inhibition, with full activity restored upon removal of the inhibitor. MHPP exhibited dose-dependent, non-competitive, slow-onset inhibition of ammonia oxidation, independent of ammonium levels. By contrast, MBOA functioned as a fast-acting, uncompetitive inhibitor, with its inhibitory effect increasing with higher ammonium concentrations and MBOA doses. These results demonstrate that the efficacy of BNIs depends on compound structure, revealing distinct modes of inhibition and substrate dependencies. This study enhances our mechanistic understanding of inhibitory modes of BNI in AOB, providing a foundation for future studies with diverse nitrifiers.

## Teaser

Different plant-derived nitrification inhibitors display contrasting modes of inhibition of nitrifier respiration, linked to ammonia oxidation, in two species of ammonia-oxidizing bacteria.

## Introduction

Ammonia-oxidizing micro-organisms are pivotal drivers of the terrestrial nitrogen (N) cycle, but their activity also contributes significantly to nitrous oxide (N_2_O) emissions, a potent greenhouse gas linked to agricultural practices. Nitrification, the microbial oxidation of ammonia (NH_3_) to nitrate (NO_3_^–^), is a key process underpinning these emissions, especially in aerobic environments. Agricultural soils account for approximately 73% of global anthropogenic N_2_O emissions, with microbial nitrification and associated pathways responsible for up to 50% of fertilizer-derived N loss (Kuypers et al. 2018, Sutton et al. ). Consequently, strategies to suppress nitrification are increasingly recognized as critical for improving nitrogen use efficiency and mitigating greenhouse gas emissions in agroecosystems.

Biological nitrification inhibitors (BNIs) are plant-derived compounds that suppress ammonia-oxidizer activity. BNI has emerged as a promising, sustainable approach to regulating soil nitrification (Subbarao et al. 2009). BNIs have been identified in key crop species such as sorghum, maize, wheat, and rice (Zakir et al. 2008, Lu et al. 2019, Nardi et al. 2020, Subbarao et al. 2021, Otaka et al. 2023). Notable compounds include methyl 3-(4-hydroxyphenyl)propionate (MHPP) from sorghum (Zakir et al. 2008), 6-methoxybenzoxazolinone (MBOA) from maize and wheat (Mwendwa et al. 2021, Otaka et al. 2023), and gallic acid (GA), a common component of root exudates (Rice and Pancholy 1973). Some BNI compounds, like MHPP, are thought to specifically inhibit ammonia monooxygenase (AMO), which is the key enzyme catalyzing the first step of ammonia oxidation, whereas other compounds, such as MBOA, sorgoleone, and GA, affect broader physiological pathways in both micro-organisms and plants (Nardi et al. 2020, Otaka et al. 2023).

Three major groups of chemolithoautotrophic nitrifiers—ammonia-oxidizing bacteria (AOB), ammonia-oxidizing archaea (AOA), and complete ammonia oxidizers (comammox)—mediate ammonia oxidation in soils. AOB typically dominate under high ammonium and neutral pH conditions common in fertilized soils (Hink et al. 2018, Prosser et al. 2020). Ammonia oxidation by AOB proceeds via three sequential enzymatic reactions: (i) AMO-mediated oxidation of NH_3_ to hydroxylamine (NH_2_OH); (ii) hydroxylamine oxidoreductase (HAO)-mediated conversion of NH_2_OH to nitric oxide (NO); and (iii) subsequent oxidation of NO to nitrite (NO₂⁻) by an as yet unidentified enzyme (Caranto and Lancaster 2017). The two most extensively studied AOB strains are *Nitrosomonas europaea* and *Nitrosospira multiformis. N. europaea* is frequently used as a model organism due to its ease of cultivation (Brandt et al. 2001, Subbarao et al. 2009, Kaur-Bhambra et al. 2022), though it is more representative of high-nitrogen, wastewater environments (Wells et al. 2009). In contrast, *N. multiformis*, though lesser studied, has been used as a representative model of AOB in several soil ecosystems (Brandt et al. 2001, Shen et al. 2013, Kaur-Bhambra et al. 2022).

Given its central role and broad substrate specificity, AMO is a prime target for chemical inhibitors (Hooper et al. 1997). Inhibitors may exhibit competitive, noncompetitive, uncompetitive, or mixed modes of action, each differentially altering the kinetic parameters, Michaelis constant (*K*_m_), which reflects the enzyme-substrate affinity, and maximal enzymatic velocity (*V*_max_) differently (Whiteley 2000). Competitive inhibitors structurally resemble the substrate and compete for binding at the enzyme’s active site, as in the case of methane working as a competitive inhibitor of AMO (Keener and Arp 1993). Competitive inhibitors increase the *K*_m_ due to reduced enzyme-substrate affinity, but do not alter the *V*_max_. Uncompetitive inhibitors bind exclusively to the enzyme–substrate complex, stabilizing it in an inactive form, resulting in a proportional simultaneous reduction of both *K*_m_ and *V*_max_. Non-competitive inhibitors bind to allosteric sites on either the free enzyme or the enzyme–substrate complex, thereby reducing *V*_max_ with variable effects on *K*_m_. Mixed-mode inhibition combines features of both competitive and non-competitive inhibition, where the inhibitor can bind both to the free enzyme and to the enzyme–substrate complex, with differing affinities. This results in changes to both *K*_m_ and *V*_max_, often in an unbalanced manner. Complex hydrocarbons like halogenated alkanes have been shown to cause mixed or noncompetitive inhibition of AMO by structural interference (Keener and Arp 1993) (Fig. S1). Enzyme inhibition can be either reversible, as for the modes of inhibition described above, or irreversible. Irreversible inhibition can occur when inhibitors bind covalently to the enzyme’s active site, thereby permanently inactivating the enzyme. For instance, acetylene and dimethylsulfide act as irreversible AMO inhibitors through covalent modification (Hyman and Wood 1985, Juliette et al. 1993), whereas thiourea derivatives inhibit AMO by chelating its copper cofactors (Hyman et al. 1990, McCarty 1999). Structurally diverse BNIs have been described (Nardi et al. 2020), but their modes of action have so far not been evaluated. Hence, it remains unclear if they act through active site binding, allosteric modification, or complex substrate interactions.

This study addresses several of these knowledge gaps by using enzyme kinetic profiling of *N. europaea* and *N. multiformis* to three structurally diverse BNIs: GA, MHPP, and MBOA. This work tested the following hypotheses: H1: The selected BNIs act as reversible inhibitors of AOB ammonia oxidation, suggesting non-covalent interactions rather than irreversible enzyme inactivation. H2: BNIs exert noncompetitive modes of inhibition, consistent with mechanisms observed previously for structurally complex polycyclic inhibitors. By incorporating kinetic modelling, BNI research gains a mechanistic foundation that clarifies inhibitory modes of action, surpasses reliance on estimated activity reductions, and strengthens fundamental insight into the structure-function relationships shaping interactions between nitrifiers and BNI compounds.

## Material and methods

### Batch cultivation of ammonia-oxidizing bacteria and BNI media preparation

Two AOB strains were used: *Nitrosomonas europaea* ATCC 19718 and *Nitrosospira multiformis* ATCC 25196 (obtained from NCIMB). The strains were cultivated in filter-sterilized mineral salts medium (Donaldson and Henderson 1989) as previously described (Brandt et al. 2001) with the following modifications: 15 mM (NH_4_)_2_SO_4_ (corresponding to 30 mM of NH_4_), 40 mM 4-(2-hydroxyethyl)-1-piperazineethanesulfonic acid (HEPES), 20 mM NaHCO_3_, 0.02% phenol red, and 1 ml l^–1^ of trace elements (Verhagen and Laanbroek 1991), with the pH adjusted to 7.5 using NaOH. Cultures were maintained in the dark at 28 °C with shaking at 200 rpm in 1 l polystyrene bottles. Growth was monitored by measuring nitrite production via a colourimetric Griess assay (Shinn 1941), using NaNO_2_ standards (0–200 μM) and a microplate reader (BioTek) at 540 nm.

To validate the suitability of dimethyl sulfoxide (DMSO; CAS: 67–68–5) as a co-solvent in BNI assays, its effect on ammonia oxidation kinetics was examined. Three DMSO concentrations, 0.05% (v/v), 0.1% (v/v), and 0.2% (v/v), were tested. BNI experiments were performed with the following three BNIs: GA (CAS: 149–91–7), MHPP (CAS: 5597–50–2), and MBOA (CAS: 532–91–2) (all from Sigma–Aldrich, >99% purity), tested at the following concentrations: MHPP at 200, 300, and 400 µM; MBOA at 15, 25, 35, and 45 µM; and GA at 250 µM. The upper limits of the BNI concentration ranges were determined by their aqueous solubility and the requirement to achieve >50% inhibition of the tested AOB strains at 5 mM ammonium. The concentrations were applied at the same dose in both strains to facilitate direct comparison.

### Measurement of ammonium-dependent oxygen consumption

AOB cultures were harvested at the late exponential phase by centrifugation at 4000 × *g* for 15 min at 4°C and washed with N-free medium. Cells were resuspended in N-free medium to an optical density of OD_600_ = 0.1 (VWR VR-1200 spectrophotometer). The BNIs at tested concentrations were then added to the cell suspension medium. Ammonia oxidation rates were calculated using a stoichiometric oxygen-to-ammonia consumption ratio of 1.5 : 1 at various concentrations of BNIs using a micro-respiration (MR) system (UNISENSE, Aarhus, Denmark) following established protocols (Martens-Habbena and Stahl 2011, Jung et al. 2022). Briefly, measurements were performed in 10-ml dual-chamber glass MR systems, stirred at 200 rpm and maintained at 28°C (± 0.1°C) in the dark in a recirculating water bath. Ammonium (in the form of (NH_4_)_2_SO_4_ was added at a final concentration of 0.5–20 mM to initiate oxidation, with doses determined based on previous studies (Martens-Habbena and Stahl 2011, Jung et al. 2022). Ammonium concentrations were chosen following previous studies, with at least six concentrations included to enable robust kinetic modelling. O_2_ consumption was monitored with an Opto-MR O_2_ microoptode (UNISENSE), and O_2_ consumption rates were determined from the linear decrease (R^2^ > 0.99) in dissolved O_2_ from ∼300 to 0 µM immediately after ammonium addition.

Reaction rates for both O_2_ consumption were fitted to the Michaelis–Menten equation:


\begin{eqnarray*}
v = \frac{{{{V}_{max}}{\mathrm{\ }} \times \left[ S \right]}}{{{{K}_m} + \left[ S \right]}},
\end{eqnarray*}

where *v* is the reaction rate (pM h^–1^ cell^–1^), *V*_max_ the maximum rate (pM h^–1^ cell^–1^), [*S*] the substrate (ammonium) concentration (µM), and *K*_m_ the apparent half-saturation constant (µM). Michaelis-Menten model for each experimental condition was fit using non-linear least squares models with R^2^ ≥ 0.98 and *P* < 10^–10^.

To evaluate the mode of inhibition of nitrification inhibitors, apparent kinetic parameters, *V*_max(app)_ and *K*_m(app)_, were estimated in the presence of DMSO, MHPP, and MBOA at varying concentrations. Statistical comparisons of parameter estimates were performed separately for each inhibitor using one-way ANOVA, followed by Tukey post hoc tests to assess pairwise differences. Enzyme inhibition patterns were further characterized through Lineweaver–Burk transformations to further distinguish potential modes of inhibition. All statistical analyses were conducted in R (version 4.4.0) using the minpack.lm, gofedf, boot, and dplyr packages. Chemical stability of inhibitors in the assays is detailed in the Supplementary data.

### Cell counting

Cell concentrations were determined by DAPI staining. Briefly, 100 µl of culture was incubated with 2 µg DAPI for 10 to 15 min at 4°C in the dark. Stained cells were filtered onto 0.22 µm nitrocellulose filters, rinsed with 5 ml nitrogen-free mineral medium, air-dried, mounted onto microscope slides, and imaged under an Olympus BX60 fluorescence microscope (400 × magnification). Cell counts were conducted in triplicate using ImageJ software (Schneider et al. 2012). All experiments in the micro-respirometry set-up showed consistent cell densities, with approximately 4×10^6^ cells ml^–1^ for N. europaea and 5×10^6^ cells ml^–1^ for *N. multiformis*.

### Inhibition effect calculation

To assess if ammonium concentration influences the efficacy of BNIs, inhibition was quantified for each combination of ammonium and inhibitor concentration for each AOB strain. Inhibition was calculated based on the reduction in O_2_ consumption using the following equation:


\begin{eqnarray*}
\textit{Inhibition}\ \left( \% \right) = 100\ - \left( {\frac{{{{{\mathrm{O}}}_2}{\mathrm{\ consumptio}}{{{\mathrm{n}}}_{{\mathrm{\ with\ inhibitor}}}}}}{{{{{\mathrm{O}}}_2}{\mathrm{\ consumption}}{{{\mathrm{\ }}}_{{\mathrm{control}}}}}}} \right) \times 100.
\end{eqnarray*}

Control is treatment without an inhibitor. Statistical analysis of inhibition across ammonium concentrations was performed using ANOVA, followed by post-hoc tests in R to determine significant differences.

### Reversible vs irreversible inhibitor test

To assess inhibitor reversibility, *N. europaea* and *N. multiformis* were harvested at late exponential phase, resuspended at OD_600_ = 0.1, and incubated in N-free medium supplemented with 250 µM GA, 200 µM MHPP, or 50 µM MBOA for 60 mins at 28°C. Post-incubation, cells were centrifuged (4000 × *g*, 15 min, 4°C) to remove the inhibitors and resuspended in inhibitor-free N-free medium (OD_600_ ∼0.085). The obtained inhibitor-treated, washed cells were transferred to micro-respiration chambers, and the addition of 6 mM (NH_4_)_2_SO_4_ (corresponding to 12 mM NH_4_^+^) initiated O₂ consumption. Controls included: (i) untreated, unwashed cells (i.e. cells incubated without inhibitors and not centrifuged), (ii) untreated, washed cells (i.e. cells incubated without inhibitors and centrifuged), and (iii) inhibitor-treated, unwashed cells. Recovery of ammonia oxidation activity after washing was used to assess whether inhibition was reversible, with complete or near complete activity recovery suggesting that the inhibitor is reversible, while no recovery in activity suggests that the inhibitor is irreversible. O_2_ consumption rates were calculated as a linear rate of decrease in dissolved O_2_ (µM h^–1^) from ∼300 to 0 µM immediately after ammonium addition (R^2^ > 0.98). For assays involving GA, the intrinsic abiotic O_2_ consumption rate of the compound was determined and subtracted from the measured rates to obtain biologically relevant values. Changes in O_2_ consumption rates were compared between the controls and the BNI-treated (washed and unwashed) cells using ANOVA, followed by Tukey post-hoc analyses in R to determine statistical significance.

## Results

### BNI stability and oxygen scavenging activity in the assay

To ensure assay reliability, chemical stability and potential oxygen-scavenging effect, the effects of the three BNI compounds—MHPP (200 µM), MBOA (25 µM), and GA (250 µM)—were evaluated in the presence (biotic) and absence (abiotic) of AOB cells. No significant compound degradation was observed over the assay duration in either the presence or absence of cells (Table S1). The concentrations were selected arbitrarily for each compound, and no further degradation testing was conducted for MHPP and MBOA, as no effects were observed at lower concentrations. While MHPP and MBOA exhibited no measurable impact on dissolved O₂ levels in sterile medium, GA consistently reduced O₂ concentrations, indicating an intrinsic, abiotic oxygen-scavenging property (Fig. S2). Consequently, O₂ measurements in the presence of GA would be expected to show higher rates and greater variability.

### DMSO impact on ammonia oxidation kinetics

Michaelis–Menten analyses of ammonia oxidation revealed minimal physiological differences between the two tested strains (Table S2; Fig. S3; R^2^ ≥ 0.98). *Nitrosospira multiformis* exhibited a significantly higher *K*_m(app)_ than *N. europaea* (ANOVA, strain effect, *P* = 1 × 10^–4^) with values remaining within the known physiological range for *N. europaea* yet appearing to be ∼10-fold higher than the known *K*_m(app)_ for other *Nitrosospira* species (Jung et al. 2022). Meanwhile, the *V*_max(app)_ of *N. multiformis* was significantly lower than that of *N. europaea* (ANOVA, strain effect, *P* = 2 × 10^–6^). This observation is particularly significant given the limited availability of kinetic data for *Nitrosospira* species, despite their ecological prominence in terrestrial systems (Prosser and Nicol 2012, Aigle et al. 2019).

The inhibitory effect of DMSO followed Michaelis–Menten kinetics in both AOB strains (Fig. S4A-B). DMSO significantly reduced both *V*_max(app)_ and *K*_m(app)_ across both AOB strains (Fig. S4C-F; ANOVA, DMSO effect, *P* < 1.7×10^–6^ and *P* < 4.5×10^–4^, respectively). Both enzyme kinetic parameters decreased ∼1.5 fold, as the DMSO concentration increased from 0% to 0.1%, a concentration previously observed not to affect the growth of the same two AOB species (Kaur-Bhambra et al. 2022). Notably, *N. multiformis* exhibited a greater sensitivity to DMSO exposure than *N. europaea*, consistent with earlier studies demonstrating *N. multiformis* to be more sensitive to a range of toxicants than *N. europaea* (Brandt et al. 2001, Kaur-Bhambra et al. 2022). To avoid these confounding effects of DMSO, all subsequent BNI experiments were conducted in DMSO-free conditions. This, however, limited the testing range for BNIs due to the lower solubility of these compounds in water, especially MBOA.

### Reversibility of BNI-mediated inhibition

**Figure 1:**
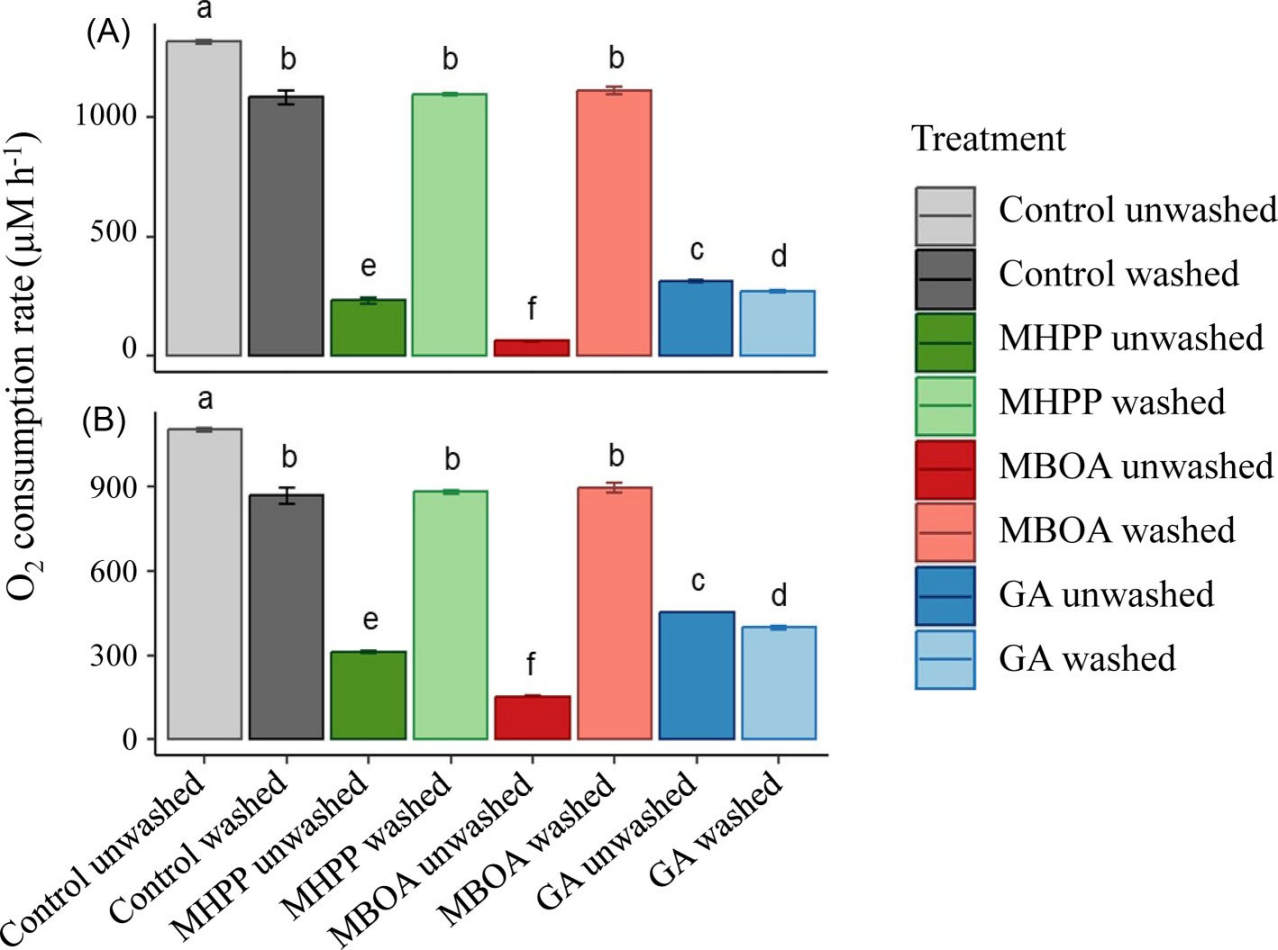
Reversibility of biological nitrification inhibition in ammonia-oxidizing bacteria. O_2_ consumption rates in *N. europaea* (Panel A) and *N. multiformis* (Panel B) following exposure to BNIs—methyl 3-(4-hydroxyphenyl)propionate (MHPP), gallic acid (GA), and 6-methoxy-2-benzoxazolinone (MBOA). Cells were treated with each BNI compound, or without for corresponding controls, for 60 min and then either washed to remove BNIs (Treatment: washed) or left unwashed (Treatment: unwashed). Means ± standard errors of the means (*n* = 3) are shown. Different letters on top of each bar denote significantly different (*P* < 0.05) O_2_ consumption rates within each test species.

The reversibility of BNI effects was evaluated by comparing cellular activity, measured as O_2_ consumption rates, between washed and unwashed cells. In cells treated with MHPP and MBOA, washing (i.e. removal of inhibitors) restored O_2_ consumption rates to 99% of the corresponding washed controls in both *N. europaea* (Fig. 1A) and *N. multiformis* (Fig. 1B), indicating that inhibition by these compounds was fully reversible. Notably, full recovery was observed regardless of the duration of prior inhibitor exposure (60 or 120 min) (Fig. S5). In contrast, cells exposed to GA did not recover following washing. Instead, O_2_ consumption rates further declined by 24% even after GA removal, suggesting a persistent inhibitory effect (Fig. 1, Fig. S5). Prolonged exposure to GA for 120 min resulted in near-total inhibition of cellular respiration in washed cells of both test strains (Fig. S5). These findings indicate that GA causes irreversible inhibition of ammonia oxidation. Washing via centrifugation caused a 15% decrease in O_2_ consumption relative to unwashed controls in both strains (Fig. 1).

### Effect of pre-incubation time on apparent enzyme kinetic parameters (*V*_max(app)_ and *K*_m(app)_)

**Figure 2:**
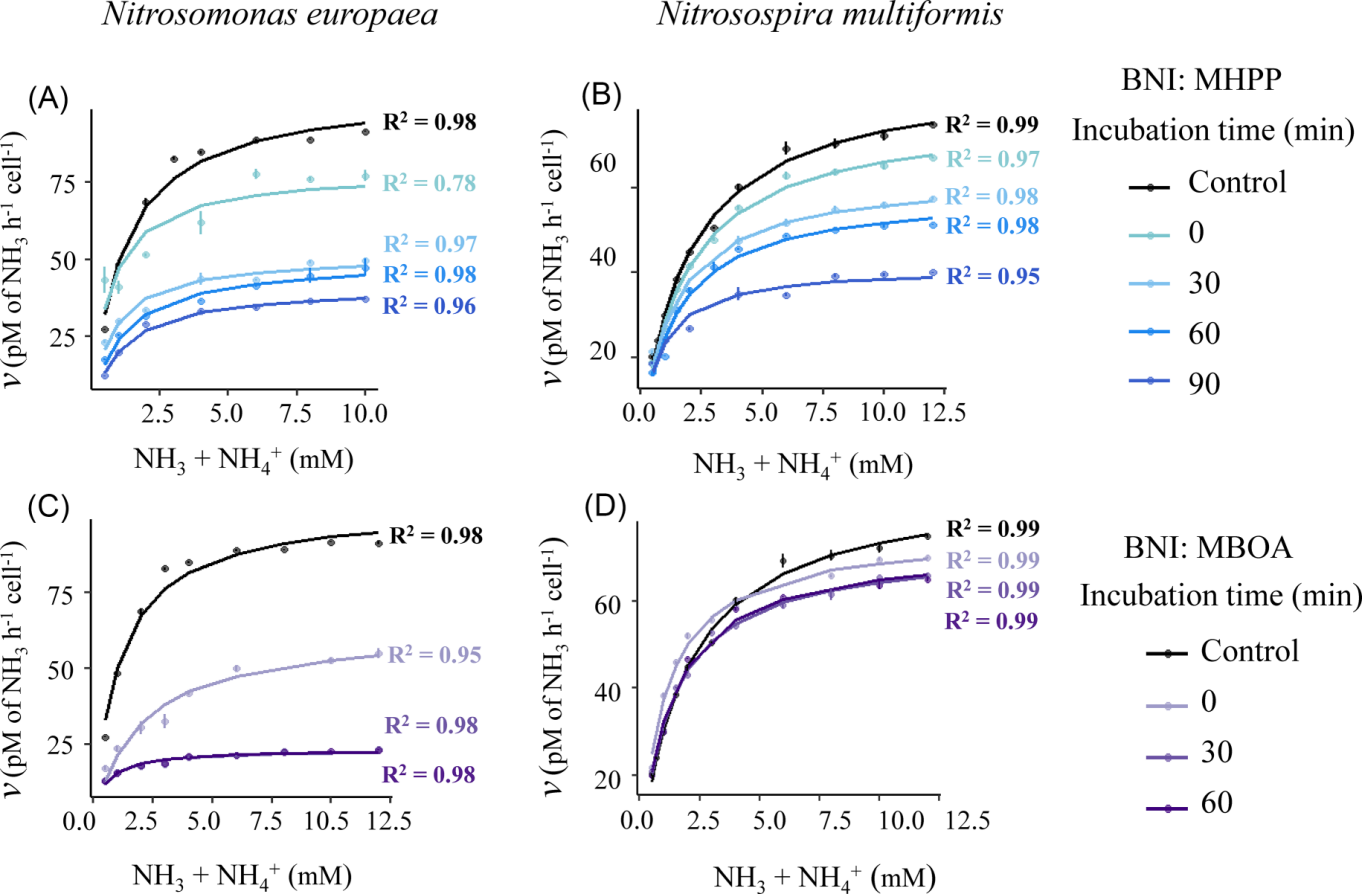
Influence of pre-incubation time with BNIs on enzyme kinetics in ammonia-oxidizing bacteria. Michaelis–Menten plots showing ammonium consumption rates and the corresponding model R² values with 200 µM 3-(4-hydroxyphenyl)propionate (MHPP) in *N. europaea* (Panel A) and *N. multiformis* (Panel B), and with 6-methoxy-2-benzoxazolinone (MBOA) in *N. europaea* (Panel C; 35 µM MBOA) and *N. multiformis* (Panel D; 15 µM MBOA). Error bars represent standard errors of the means (*n* ≥ 3).

O₂ consumption kinetics in the presence of BNI compounds depended on the pre-incubation time, reflecting the time needed for the studied BNIs to cause inhibition of cellular respiration. For reliable kinetic analysis, cells required a 30–60 min pre-incubation with inhibitors to achieve near-perfect linear O_2_ consumption (R^2^ ≥ 0.999), whereas reduced pre-incubation times led to increasing departures from linearity (Table S3). These deviations from linearity also resulted in poor fits to the Michaelis–Menten model which would have subsequently confounded the interpreted kinetics (Fig. 2). To improve data consistency and model reliability, cells were therefore pre-incubated with BNI compounds in the absence of ammonium for varying durations: 0, 30, 60, and 90 min for MHPP (200 µM), and 0, 30, and 60 min for MBOA (35 µM for *N. europaea* and 15 µM for *N. multiformis*) (Fig. 2). Pre-incubation for 60 min with MHPP and 30 min with MBOA significantly improved the linearity of O_2_ consumption (R^2^ ≈ 1) (Table S3) and the fits of the Michaelis–Menten model (Fig. 2), bringing both in line with control treatments and thereby validating the pre-incubation approach.

In the case of MHPP, *V*_max(app)_ progressively decreased with increasing pre-incubation in both AOB strains (Fig. S6). *K*_m(app)_ was also affected by pre-incubation time, but no consistent trend was observed for the time effect (Fig. S6). Lineweaver–Burk analysis revealed a shift in mode of inhibition with increasing pre-incubation time, transitioning from uncompetitive to predominantly non-competitive inhibition (Fig. S7). For MBOA, both strains exhibited significant changes in *K*_m(app)_ and *V*_max(app)_ within 30 min of pre-incubation; no further changes in kinetic parameters occurred with extended pre-incubation (Fig. 2, Figs S6-Fig. S7).

Collectively, our results demonstrate that the determination of apparent enzyme kinetic parameters was very sensitive to the choice of pre-incubation time. Based on the absence of further changes in inhibition kinetics in the Lineweaver–Burk analyses and the consistently high fit quality (R^2^ ≥ 0.99), we selected pre-incubation times of 60 min for MHPP and 30 min for MBOA for all subsequent dose–response assays described below.

### Dose-dependent effects of BNIs on enzyme kinetics

**Figure 3:**
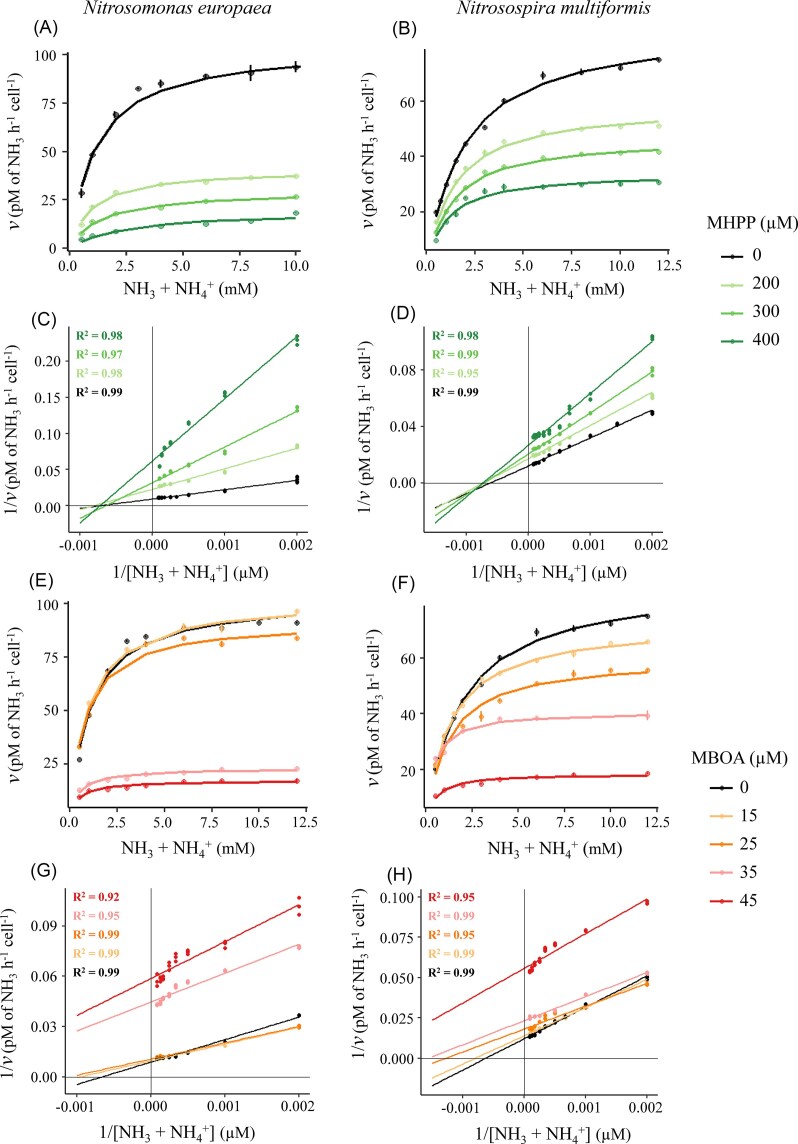
Impacts of BNIs on enzyme kinetics in ammonia-oxidizing bacteria. Michaelis–Menten plots showing ammonium consumption rates in the presence of varying concentrations of 3-(4-hydroxyphenyl)propionate (MHPP) with 60 min pre-incubation in *N. europaea* (Panel A) and *N. multiformis* (Panel B) with corresponding Lineweaver–Burk plots in Panels C and D, respectively. Michaelis–Menten plots showing ammonium consumption rates in the presence of varying concentrations of 6-methoxy-2-benzoxazolinone (MBOA) with 30 min pre-incubation in *N. europaea* (Panel E) and *N. multiformis* (Panel F) with corresponding Lineweaver–Burk plots in Panels G and H, respectively. Data are presented as dot plots with fitted curves and the corresponding model R² values for Lineweaver–Burk plots. The R² values for Michaelis–Menten models were ≥ 0.98. Error bars represent standard errors of the mean (*n* ≥ 3).

**Figure 4:**
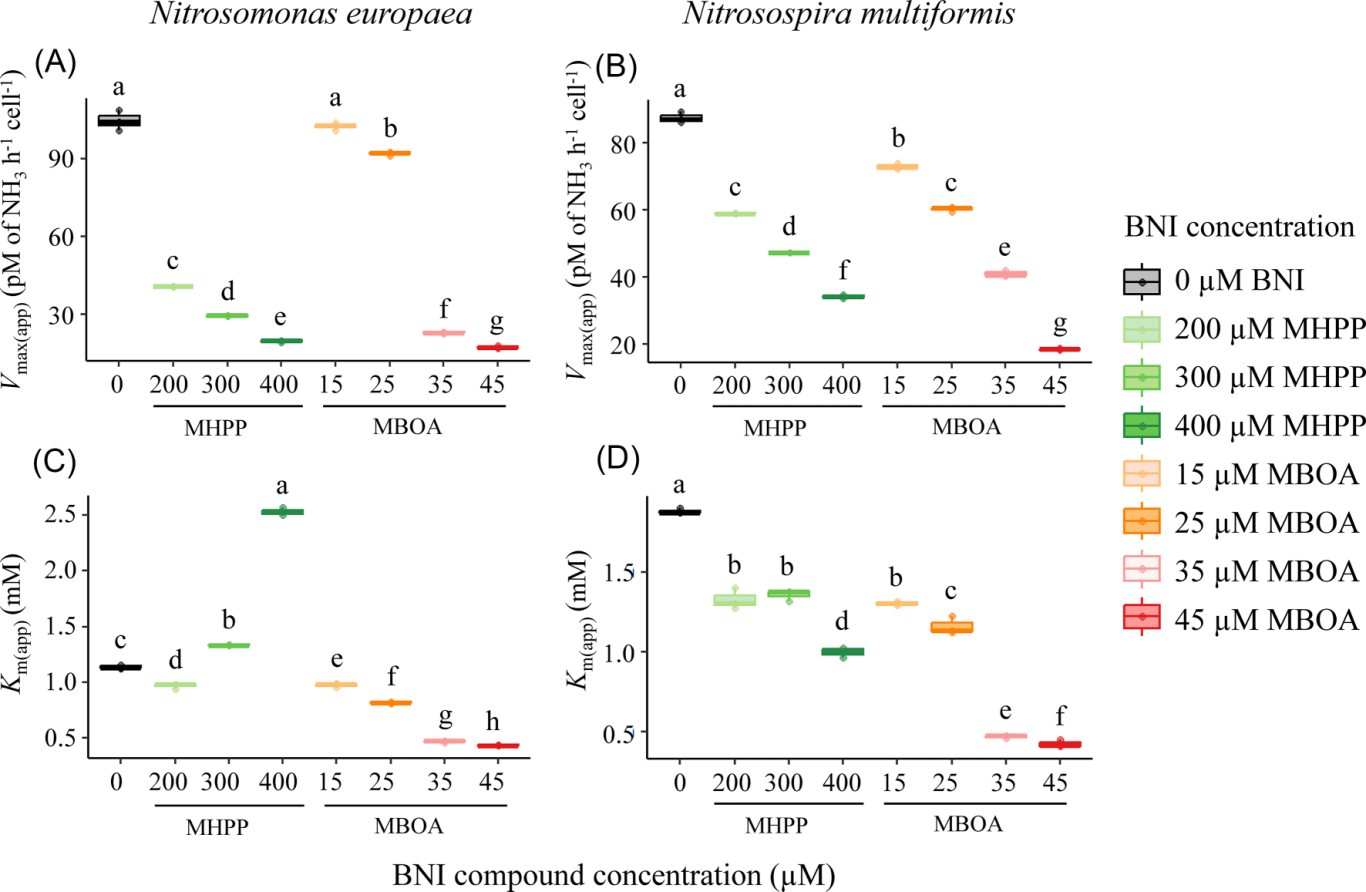
Dose-response effects of BNIs on the enzyme kinetic parameters in ammonia-oxidizing bacteria. Maximal enzymatic velocity, *V*_max(app)_, across varying doses of 3-(4-hydroxyphenyl)propionate (MHPP; 60 min pre-incubation) and 6-methoxy-2-benzoxazolinone (MBOA; 30 min pre-incubation) in *N. europaea* (Panel A) and *N. multiformis* (Panel B). Enzyme saturation constant, *K*_m(app)_, across varying doses of 3-(4-hydroxyphenyl)propionate (MHPP; 60 min pre-incubation) and 6-methoxy-2-benzoxazolinone (MBOA; 30 min pre-incubation) in *N. europaea* (Panel C) and *N. multiformis* (Panel D). Data are presented as box plots, and different letters on top of each box denote significant differences (*P* < 0.05) in enzyme kinetic parameters within each test species.

The impacts of BNI compounds were characterized by testing the inhibitory effects of MHPP and MBOA at a range of ammonium concentrations in the AOB strains *N. europaea* and *N. multiformis*, with both BNIs exhibiting clear dose-dependent effects on enzyme kinetics (Fig. 3A–B and E–F) and apparent enzyme kinetic parameters (Fig. 4). Compared to MHPP, MBOA exerted inhibitory effects at substantially lower concentrations. MHPP progressively reduced *V*_max(app)_ in both strains from 104.7 ± 1.8 to 19.8 ± 0.2 pM h^–1^ cell^–1^ in *N. europaea* and from 87.3 ± 0.8 to 34.5 ± 0.3 pM h^–1^ cell^–1^ in *N. multiformis* (Fig. 4A–B). Likewise, MBOA progressively reduced *V*_max(app)_ in both strains from 104.7 ± 1.8 to 17.5 ± 0.3 pM h^–1^ cell^–1^ in *N. europaea* and from 87.3 ± 0.8 to 18.5 ± 0.1 pM h^–1^ cell^–1^ in *N. multiformis* (Fig. 4A–B). Impacts of BNIs on *K*_m(app)_ for ammonium were more variable (Fig. 4C-D). *K*_m(app)_ values generally decreased with increasing MBOA dose in both *N. europaea* and *N. multiformis*; surprisingly, MHPP affected *K*_m(app)_ differently in the two test species. Hence, *K*_m(app)_ values generally decreased with increasing MHPP dose in *N. multiformis*, whereas *K*_m(app)_ values increased at the highest concentrations tested for MHPP in *N. europaea*.

Our data indicate contrasting modes of inhibition for MHPP and MBOA. Hence, Lineweaver–Burk analyses indicated a mixed non-competitive inhibition of MHPP for both strains (Fig. 3C–D), whereas the progressively decreased *K*_m(app)_ and *V*_max(app)_ with increasing MBOA concentrations in both test strains is consistent with uncompetitive inhibition. Parallel shifts in Lineweaver–Burk plots further corroborated an uncompetitive inhibition mechanism (Fig. 3G–H). In Lineweaver–Burk analysis, at lower MBOA concentrations (15–25 µM), regression lines intersected with control lines in the first quadrant due to a hormesis effect. Hence, low doses of inhibitor enhanced enzymatic activity, which occurred at low ammonium concentrations in this instance (Fig. 3G–H). At higher concentrations (35–45 µM), the regression lines shifted in parallel without intersection, consistent with an uncompetitive inhibition model (Fig. 3G–H).

### Effect of ammonium concentration on inhibitor efficiency

**Figure 5:**
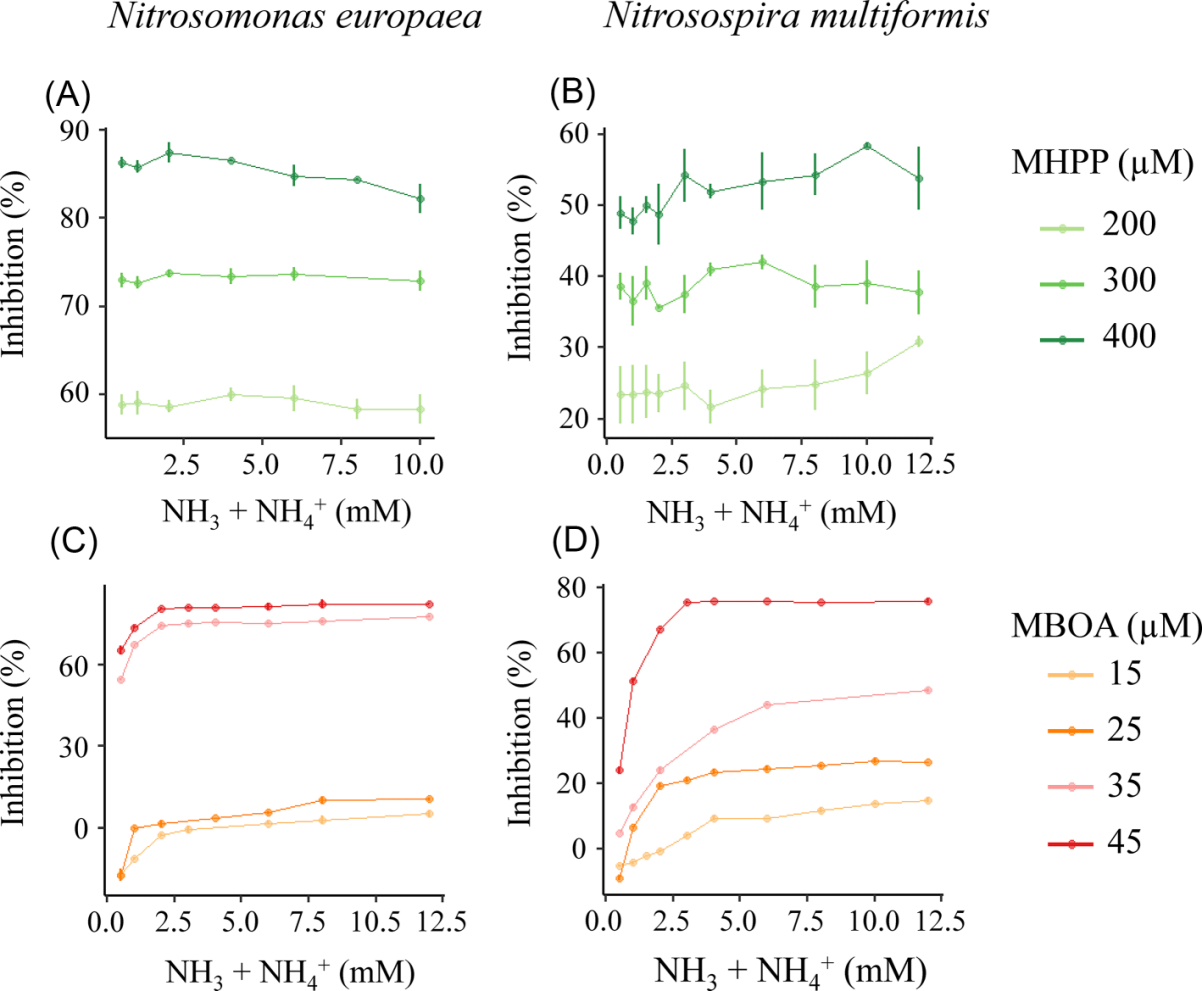
The effect of ammonium concentration on the inhibitory efficiency of BNIs in ammonia-oxidizing bacteria. Inhibition of O₂ respiration rates at different ammonium concentrations as caused by 3-(4-hydroxyphenyl)propionate (MHPP; 60 min pre-incubation) in *N. europaea* (Panel A) and *N. multiformis* (Panel B), and 6-methoxy-2-benzoxazolinone (MBOA; 30 min pre-incubation) in *Nitrosomonas europaea* (Panel C) and *N. multiformis* (Panel D). Error bars represent standard errors of the means (*n* ≥ 3).

To evaluate whether ammonium concentration influences BNI efficiency, we measured O₂ consumption rates and calculated inhibition across a range of ammonium concentrations. MHPP-mediated inhibition of cellular respiration was not significantly affected by ammonium concentration in *N. europaea* (ANOVA, ammonium effect at each MHPP concentration, *P* > 0.09) (Fig. 5A) or *N. multiformis* (ANOVA, ammonium effect at each MHPP concentration, *P* > 0.52) (Fig. 5B). In contrast, MBOA inhibition efficiency increased significantly with increasing ammonium concentration in both *N. europaea* (ANOVA, ammonium effect at each MBOA concentration, *P* < 2.9×10^–12^) (Fig. 5C) and *N. multiformis* (ANOVA, ammonium effect at each MBOA concentration, *P* < 2.0×10^–16^) (Fig. 5D). These patterns suggest that MHPP likely targets the AMO enzyme irrespective of its catalytic turnover, whereas MBOA, potentially by impacting enzyme-substrate complex formation, interferes more effectively under conditions of high substrate availability.

## Discussion

This study reveals that structurally distinct biological nitrification inhibitors exert fundamentally different modes of inhibition on AOB, reflecting divergent underlying biochemical interactions. By resolving enzyme-kinetic responses across compounds, we show that BNIs differ not only in potency but also in reversibility, time dependence, and substrate interactions. In contrast to H1, GA was identified as an irreversible inhibitor of ammonia oxidation (Fig. 1). Under alkaline conditions, GA functions as an O_2_ scavenger (Fig. S2; this study, Pant et al. 2019) This capacity arises from auto-oxidation of its phenolic groups, likely further accelerated by trace metals in the growth medium, leading to rapid depletion of dissolved O_2_. However, given that AOB retains activity even at sub-micromolar O_2_ concentrations, O_2_ depletion alone is unlikely to fully account for the observed irreversible inhibition, especially once GA is washed out (Bellucci et al. 2011). Instead, GA may directly disrupt bacterial membranes, consistent with reports that it increases membrane permeability, triggers ion leakage, and depletes ATP in Gram-negative bacteria (Lu et al. 2016), thereby explaining the lack of recovery once the inhibitor is removed.

Consistent with these findings, GA has been reported to completely inhibit nitrification by *Nitrosomonas* and *Nitrobacter* sp. in soils and soil slurries at low concentrations, although such effects are likely transient in surface soils due to rapid leaching (Rice and Pancholy 1973). Beyond inhibiting nitrification, GA may broadly suppress soil nitrogen cycling by reducing microbial activity, ammonification, and nitrate reduction, potentially through both antimicrobial and metal-chelating effects (Borges et al. 2013, Narayanaswamy et al. 2023). While GA shows potential as a biological nitrification inhibitor, its non-selective mode of action raises concerns regarding unintended impacts on beneficial soil micro-organisms and long-term soil functioning, warranting further investigation.

Unlike GA and consistent with H1, MHPP and MBOA displayed a reversible inhibition of AOB, with full activity restored upon inhibitor removal (Fig. 1). MHPP specifically inhibits AMO enzyme complex, as MHPP inhibition is rescued in the presence of hydroxylamine, the intermediate substrate in the ammonia oxidation process (Zakir et al. 2008). In this study, MHPP displayed a time-dependent inhibition of AOB, acting as a slow-binding, mixed-type inhibitor of the AMO enzyme. In slow-binding inhibition, the inhibitor-enzyme complex forms through a two-step process, in which an initial weak complex gradually transitions to a more tightly bound state; as a result, the apparent inhibition kinetics change as pre-incubation time increases, reflecting the gradual establishment of the high-affinity complex (Morrison and Walsh 1988). A likely explanation for MHPP requiring longer pre-incubation periods is that initial MHPP binding perturbs AMO structure subtly, enabling a slower, secondary rearrangement that locks the inhibitor into a higher-affinity site, thereby altering substrate interaction over time. Confirming H2, prolonged exposure to MHPP shifted inhibition from mixed to predominantly non-competitive mode (Fig. S7), consistent with a slow conformational change in AMO that enhances inhibitor binding stability (Shamsudin et al. 2017, Pesaresi 2023). MHPP has been shown to inhibit the growth and activity of both AOA and AOB (Kaur-Bhambra et al. 2022, Kolovou et al. 2023), indicating that its AMO-targeted mode of action is conserved across ammonia-oxidizing micro-organisms and suggesting that comammox organisms are also likely susceptible. However, MHPP has also been reported to inhibit nitrite-oxidizing bacteria (Kolovou et al. 2023), indicating that its inhibitory effects may extend beyond ammonia oxidizers and that complete specificity cannot be assumed.

In contrast with MHPP, MBOA functioned as a fast-acting, uncompetitive inhibitor, with its inhibitory effect increasing with higher ammonium concentrations, thereby refuting H2. Unlike MHPP, MBOA’s molecular target remains undefined, as it has been shown that MBOA blocks both ammonium to nitrite (AMO + HAO enzymatic pathway) and hydroxylamine to nitrite (HAO enzymatic pathway) in *N. europaea* (Otaka et al. 2023). Our data suggest that MBOA may disrupt the electron transport chain (ETC) rather than AMO. Three lines of evidence support this hypothesis: (i) O_2_ uptake assays showed ammonium-dependent inhibition, implicating ETC-linked respiration; (ii) inhibition was concentration-dependent but not time-dependent, unlike classical slow-binding AMO inhibitors such as DCD and MHPP (Yildirim et al. 2023); and (iii) MBOA exhibits broad-spectrum allelopathic effects (Kato-Noguchi and Macías 2008, Otaka et al. 2023, Thoenen et al. 2023), consistent with targeting conserved ETC components. This mode of action parallels that of sorgoleone, a well-characterized allelochemical BNI that inhibits by mimicking ubiquinone in the ETC (Rimando et al. 1998).

Reflecting their contrasting inhibitory mechanisms, MHPP and MBOA displayed distinct, compound-specific responses to substrate availability. MHPP-mediated inhibition was independent of external ammonium concentrations (Fig. 5), indicating that its suppressive effect is resilient to fluctuations in substrate supply highlighting MHPP’s utility for long-term nitrification control in agricultural soils. In contrast, MBOA exhibited strong ammonium-dependent inhibition, with greater suppression at higher NH_4_^+^ levels (Fig. 5), suggesting that its effectiveness scales with substrate availability rather than being enhanced under limitation. This increased inhibition with substrate availability for MBOA likely explains the hormesis observed at low ammonium concentrations which might be in response to oxidative stress caused by inhibitors (Mo et al. 2025). Additionally, its efficacy under high substrate conditions makes MBOA a viable short-term inhibitor, particularly useful immediately following fertilization. However, its efficacy of both these inhibitors will vary depending on soil pH, microbial composition, and degradation (Richardson and Bacon 1995, Fomsgaard et al. 2006, Lu et al. 2019, Lan et al. 2022, Kolovou et al. 2023, Rojas-Pinzon et al. 2024). These contrasting patterns highlight that BNIs differ fundamentally in how their activity responds to ammonium supply, underscoring the need to match BNI properties with expected nutrient conditions rather than assuming uniform behaviour across compounds.

This study focused on AOB, where the two phylogenetically distinct representatives exhibited broadly similar inhibition kinetics; responses may differ substantially in other ammonia oxidizers, including AOA and comammox. AOA generally exhibit much higher substrate affinities than either AOB (Jung et al. 2022), which may render them more sensitive to certain BNIs (Kaur-Bhambra et al. 2022, Kolovou et al. 2023); however, a high strain variability in susceptibility to BNIs exists within the two groups. Similarly, AOA and AOB also show divergent susceptibility patterns to hydrocarbons due to lineage-specific differences in AMO structure and ammonia oxidation pathways (Taylor et al. 2013, Stein 2019, Wright et al. 2020, Zhao et al. 2020). These differences indicate that inhibitor responses observed in AOB cannot be directly extrapolated to AOA. Likewise, comammox organisms possess distinct AMO and downstream nitrification machinery, suggesting that their sensitivity to BNIs may differ in both magnitude and mode of action. Although the specific modes of toxic action by BNIs deserve more detailed study across phyla, our study underscores the contrasting modes of inhibition of plant-derived BNIs' interactions with AOB, with implications for their ecological roles, selectivity, and potential deployment in agroecosystems.

## Conclusion and future perspectives

This study provides new mechanistic insights into how three structurally diverse BNIs, GA, MHPP, and MBOA, inhibit AOB activity, with each exhibiting a unique mode of inhibition. GA was shown to act as a potent, irreversible inhibitor, likely through membrane damage, while MHPP demonstrated slow-onset, non-competitive inhibition of the AMO enzyme, and MBOA acted as a fast-acting, uncompetitive inhibitor, possibly targeting conserved components of the electron transport chain. These findings confirm that BNI efficacy is closely linked to compound-specific properties, including reversibility, substrate dependency, and potential enzyme targets, some of which may also be found in non-target organisms. Moreover, we observed that while MHPP inhibition was largely independent of ammonium levels, MBOA’s inhibitory effect scaled with substrate concentration, revealing contrasting ecological potentials. Future research should extend enzyme kinetic analyses of BNI to other key ammonia oxidizers, including ammonia-oxidizing archaea and comammox bacteria, which possess structurally distinct AMO enzymes and unique cellular properties that may critically influence their sensitivity to BNI compounds. Moreover, there should be a focus on evaluating the long-term ecological effects of BNIs to ensure that strategies aimed at reducing nitrogen losses do not inadvertently compromise soil health. Potential adverse effects include resistance development in nitrifier communities, side effects associated with broad-spectrum antimicrobial activity of some BNIs (e.g. GA and MBOA), and leaching of BNIs to groundwater.

## Supplementary Material

fiag032_Supplemental_File
