# Supplementary material for: Structurally diverse biological nitrification inhibitors display distinct modes of inhibition in ammonia-oxidizing bacteria
Source: FEMS Microbiol Ecol. 2026 Mar 25;102(4):fiag032. doi: 10.1093/femsec/fiag032 (PMC13056722; doi:10.1093/femsec/fiag032)
Supplement: fiag032_Supplemental_File [file fiag032_supplemental_file.docx]

**Supplementary data**

| Materials and methods | 2 |
| --- | --- |
| Results | 2 |
| Figure S1: Schematic representation of the modes of enzymatic inhibition. | 4 |
| Figure S2: O_2_ scavenging potential of BNI compounds. | 5 |
| Figure S3: Ammonium consumption kinetics of *Nitrosomonas europaea* and *Nitrosospira multiformis.* | 6 |
| Figure S4: Impact of DMSO on enzyme kinetics in *N. europaea* and *N. multiformis.* | 7 |
| Figure S5: Reversibility of enzyme inhibition. | 8 |
| Figure S6: Influence of pre-incubation time with MHPP and MBOA on apparent enzyme kinetic parameters. | 9 |
| Figure S7: Lineweaver-Burk analysis of the influence of pre-incubation time with MHPP on enzyme kinetics. | 10 |
| Table S1: Stability of BNI compounds during enzyme kinetic assay. | 11 |
| Table S2. Kinetic parameters for O₂ consumption in the AOB strains without BNI supplementation. | 12 |
| Table S3: Linearity of O₂ consumption rates. | 13 |
| Table S4: Multiple reaction monitoring (MRM) summary. | 14 |
|  |  |
|  |  |
|  |  |

**Materials and methods**

**LC-MS/MS analysis to determine stability of BNI**

The chemical stability of MHPP at 200 µM, MBOA at 25 µM, and GA at 250 µM was evaluated using liquid chromatography-tandem mass spectrometry (LC-MS/MS) under two conditions: (i) in the presence of cells with ammonium supplementation, and (ii) in the absence of cells and ammonium. Quantification was performed with an Agilent 1200 HPLC system coupled to a 4500 QTRAP triple quadrupole mass spectrometer (AB Sciex, Foster City, CA, USA).

All three compounds were diluted to 1:100 of their original concentration before measurement. For MBOA and MHPP, separation was achieved on a Kinetex biphenyl column (100 mm × 2.1 mm, 4 µm particle size, 100 Å pore size; Phenomenex, Macclesfield, U.K.) under a gradient elution program. The mobile phases consisted of 10% and 80% methanol containing 20 mM acetic acid, delivered at a flow rate of 300 µL min⁻¹ over a 25-min run. Electrospray ionisation (ESI) was employed in both positive (0–14 min) and negative (14–25 min) ionisation modes, with MHPP analysed under positive ESI and MBOA under negative ESI. Gallic acid was separated using a Kinetex RP-Polar column (100 mm × 2.1 mm; Phenomenex) under a gradient elution with mobile phases containing 7% and 78% acetonitrile, both with 20 mM acetic acid, at a flow rate of 400 µL min⁻¹ over an 8-minute run. Gallic acid was analysed in negative ESI mode.

Multiple reaction monitoring (MRM) transitions specific to each analyte are provided in Table S4. Calibration curves for each compound were generated using authentic standards across a concentration range of 0.095–200 ng mL⁻¹, and data were processed using Analyst Software 1.7 (AB Sciex). All calibrations exhibited high linearity (R² > 0.99).

The compound recovery was calculated under both abiotic and biotic conditions to evaluate the efficiency of the method.

$$Recovery \left( \% \right)= \left( \frac{Measured concentration}{Theoretical concentration} \right)\times100$$

The theoretical added BNI concentration for the assay was 200 µM for MHPP, 250 µM for GA and 25 µM for MBOA.

**List of Supplementary figures**


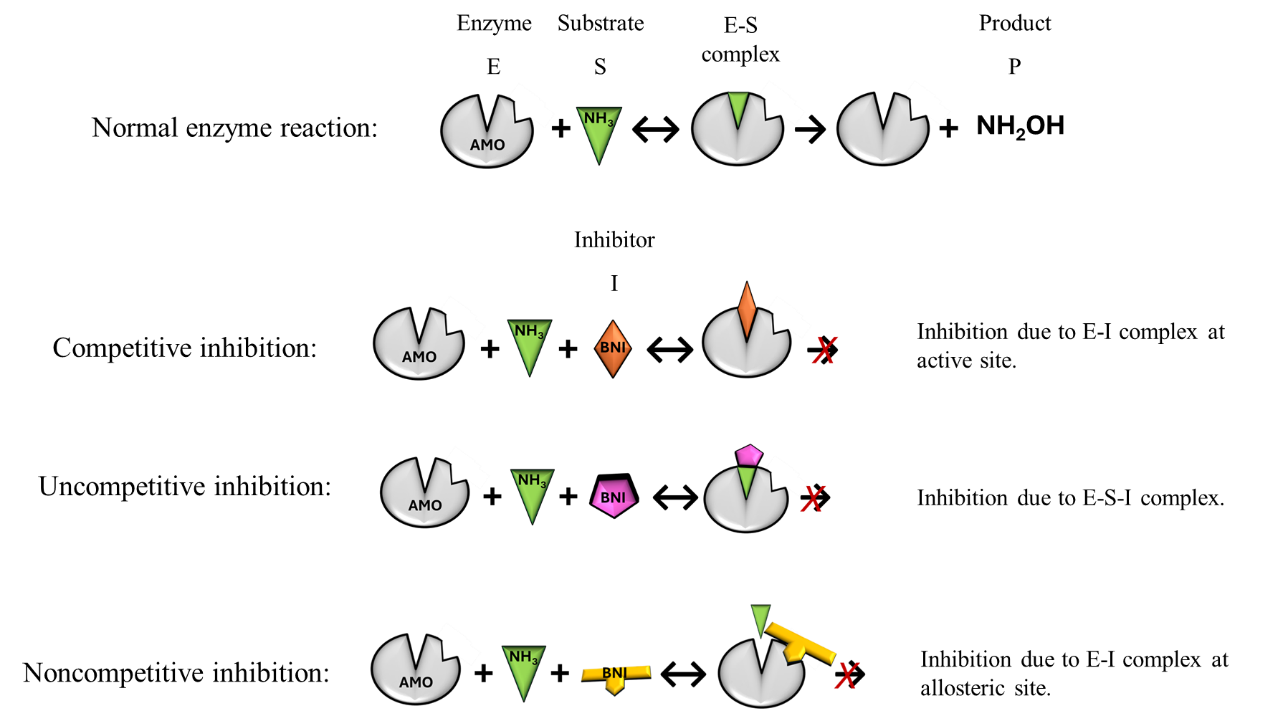


**Figure S1: Schematic representation of the modes of enzymatic inhibition.** Model created in relation to the inhibition of ammonia monooxygenase (AMO) by biological nitrification inhibitors (BNIs). In a normal ammonia oxidation pathway, AMO catalyses the oxidation of NH₃ to hydroxylamine (NH₂OH). Competitive inhibition occurs at the AMO active site by BNIs, preventing hydroxylamine production. Uncompetitive inhibitor binds only to the AMO–NH₃ complex, preventing completion of the catalytic cycle and hydroxylamine production. A noncompetitive inhibitor binds to AMO at an allosteric site, reducing enzyme activity regardless of whether ammonia is bound.


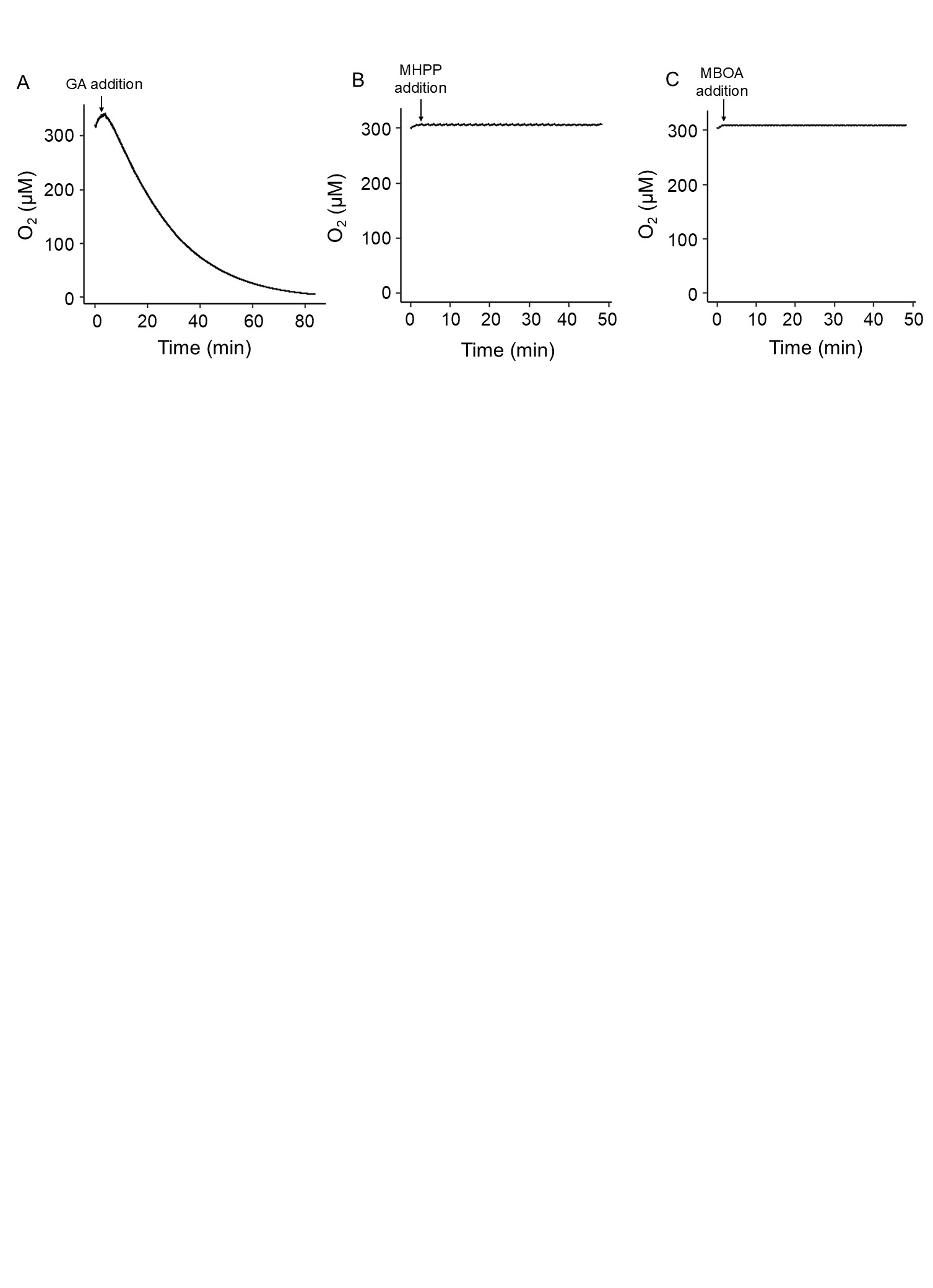


**Figure S2: O_2_ scavenging potential of BNI compounds.** Dot plot illustrating the decrease of O₂ in sterile culture media in the presence of **A.** 250 µM gallic acid (GA), **B.** 200 µM 3-(4-hydroxyphenyl)propionate (MHPP) and **C.** 25 µM 6-methoxy-2-benzoxazolinone (MBOA).


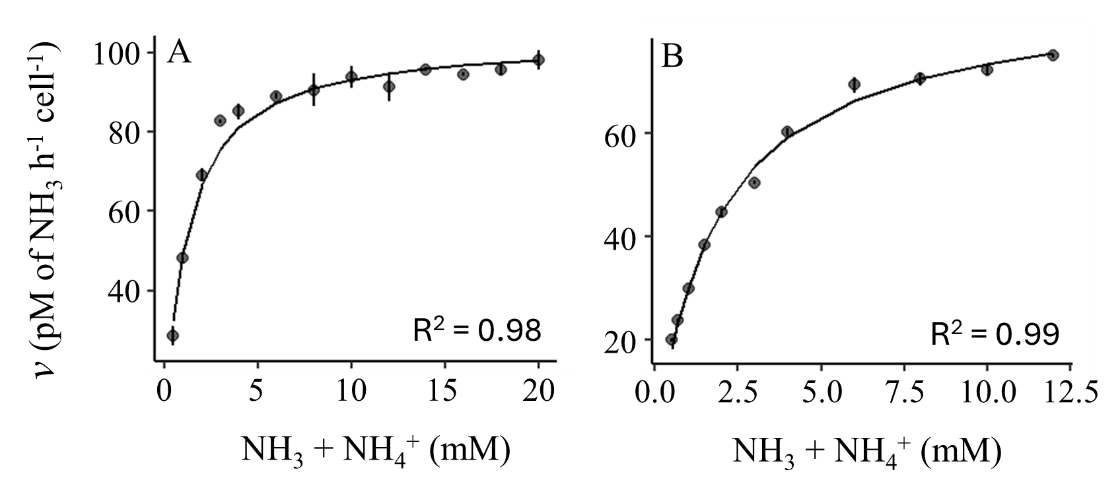


**Figure S3: Ammonium consumption kinetics of *Nitrosomonas europaea* and *Nitrosospira multiformis*.** Michaelis-Menten kinetics of ammonium consumption rates measured using O_2_ decrease in **(left to right)** *N. europaea* and *N. multiformis*. Data presented as a dot and line plot. Error bars represent standard errors of the means (n ≥ 3).


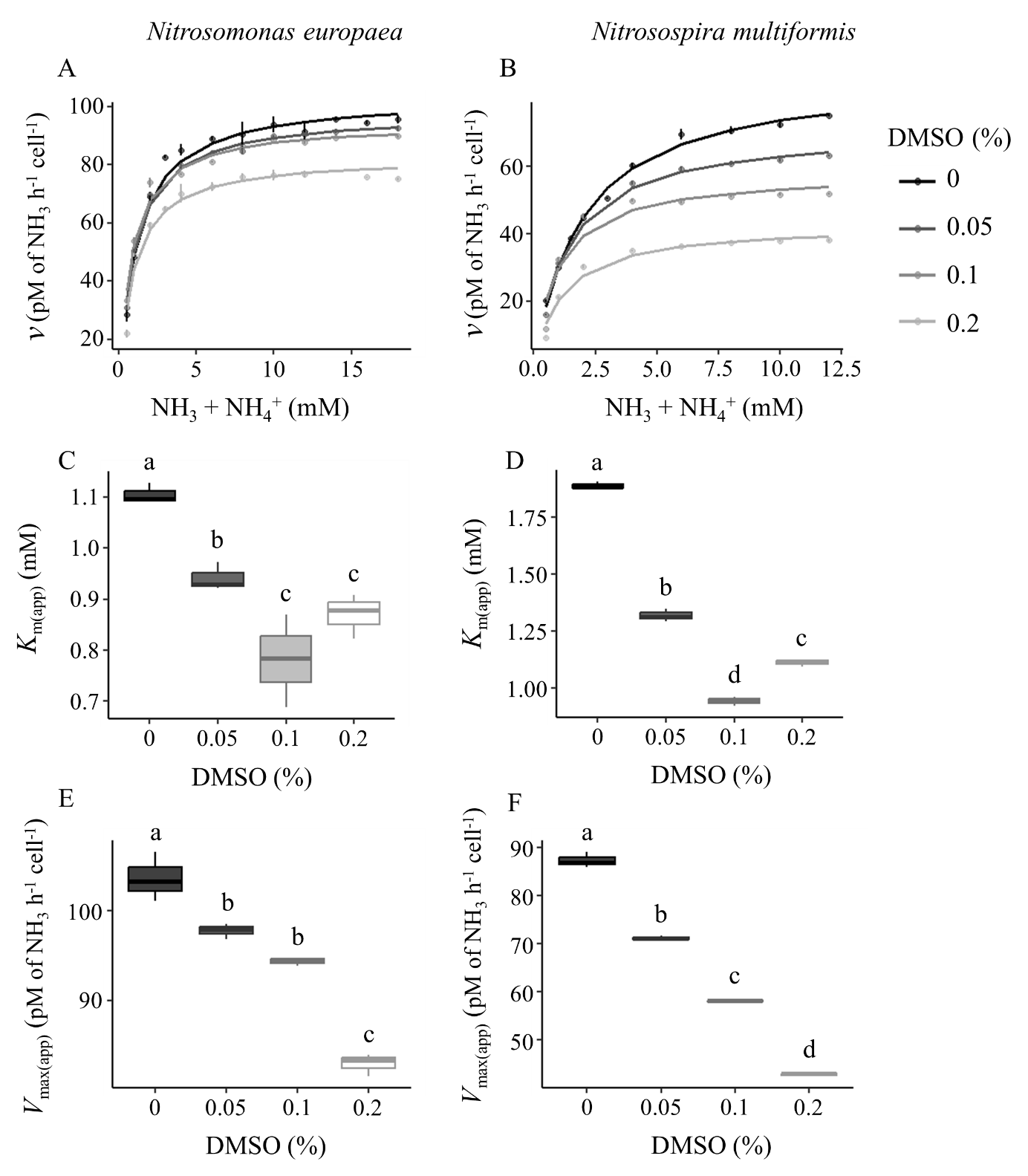


**Figure S4. Impact of DMSO on enzyme kinetics in *N. europaea* and *N. multiformis*.** **A, B,** Changes in Michaelis-Menten kinetics of ammonium consumption rates at varying dimethyl sulfoxide (DMSO) concentrations (0, 0.05, 0.1 and 0.2%) in strains **A.** *N. europaea* and **B.** *N. multiformis*. Data presented as dot and line plot. Error bars represent standard errors of the means (n ≥ 3). **C, D,** Comparison of maximal enzymatic velocity, *V*_max(app)_, across DMSO concentrations in strains **C.** *N. europaea* and **D.** *N. multiformis*. **E, F,** Comparison of enzyme saturation constant, *K*_m(app)_, across DMSO concentrations in strains **E.** *N. europaea* and **F.** *N. multiformis.* Data are presented as box plots, and different letters on top of each box plot denote significant differences (*P* < 0.05) in enzyme kinetic parameters within each subplot when tested using ANOVA, followed by post hoc analysis.


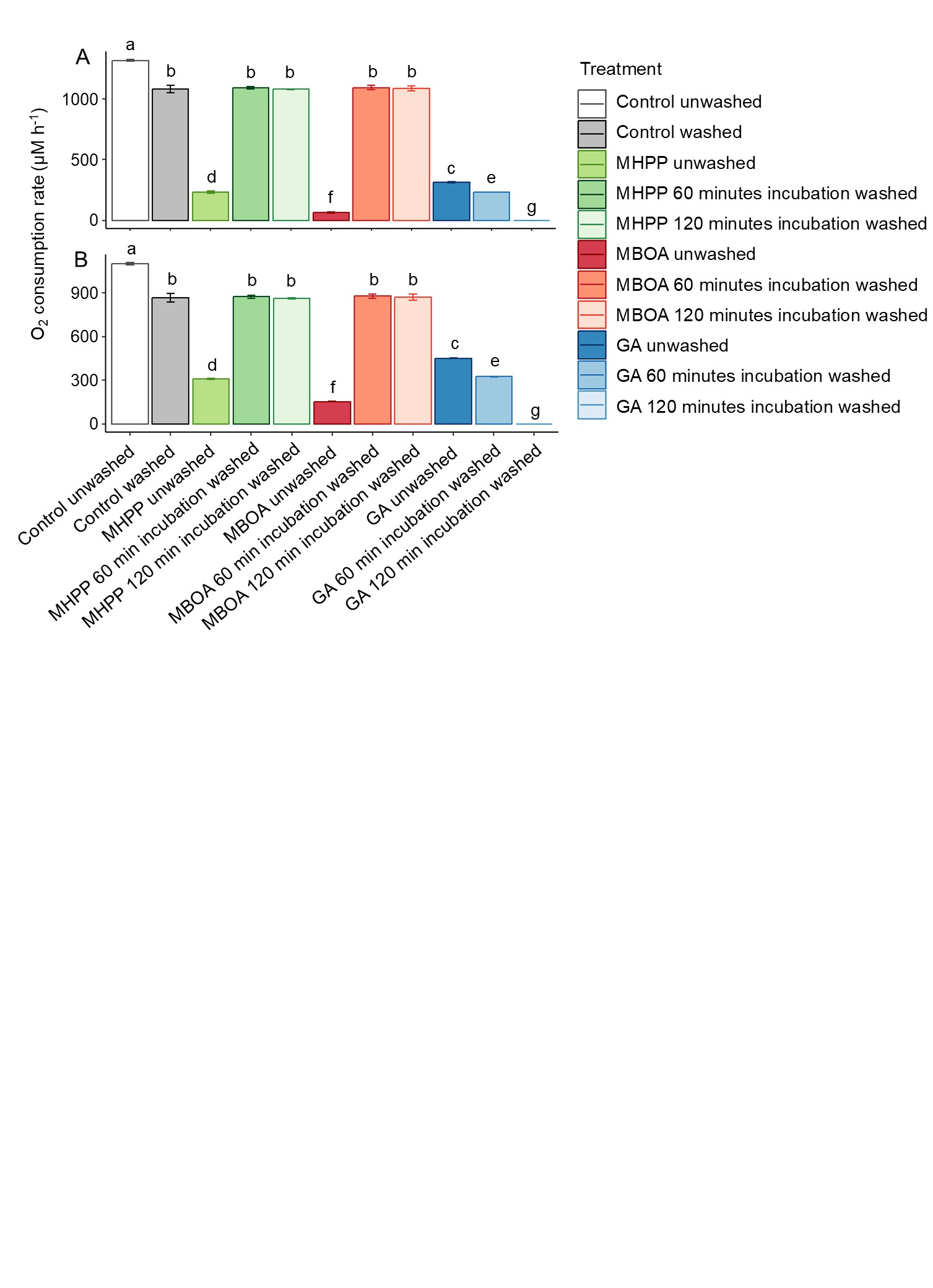


**Figure S5:** **Reversibility of enzyme inhibition.** Changes in O_2_ consumption rates after washing treatment of BNI compounds – methyl 3-(4-hydroxyphenyl)propionate (MHPP), gallic acid (GA), and 6-methoxy-2-benzoxazolinone (MBOA) in **A.** *N. europaea* and **B.** *N. multiformis*. Cells were treated with BNI compounds for 60 or 120 min and then washed to remove BNI or left unwashed for 60 mins. Control samples did not have any BNI treatment. Data are presented as bar plots. Error bars represent standard errors of the means (n = 3). Different letters on top of each bar plot denote significant differences (*P* < 0.05) in O_2_ consumption rates within each subplot when tested using ANOVA, followed by posthoc analysis.


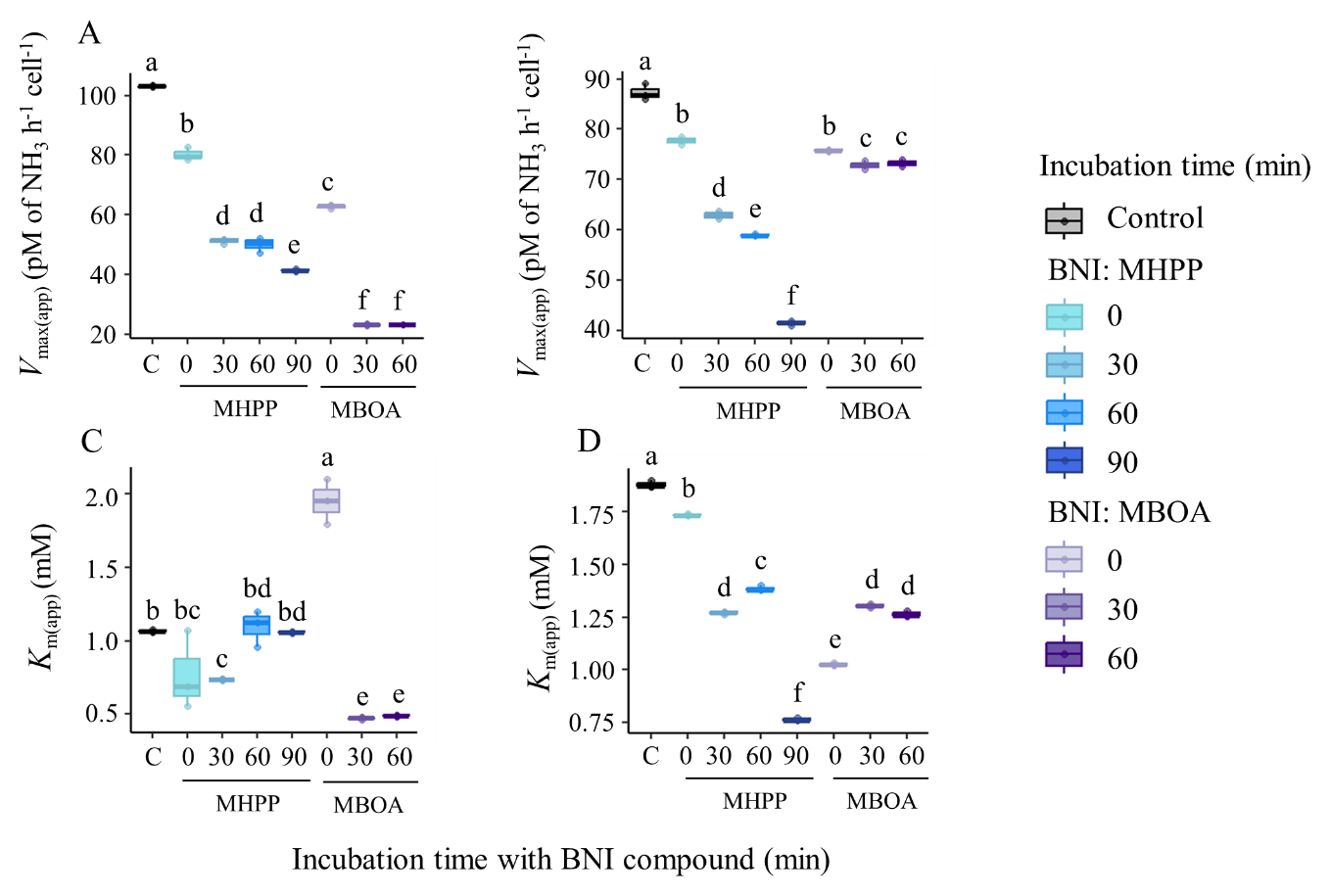


**Figure S6:** **Influence of pre-incubation time with MHPP and MBOA on the determination of apparent enzyme kinetic parameters.** Comparison of maximal enzymatic velocity, *V*_max(app)_, across varying pre-incubation times with 200 µM of 3-(4-hydroxyphenyl)propionate MHPP (pre-incubation: 0, 30, 60, and 90 min) and 6-methoxy-2-benzoxazolinone MBOA (preincubation: 0, 30, and 60 min; 35 µM in *N. europaea* and 15 µM in *N. multiformis*) in strains **A.** *N. europaea* and **B.** *N. multiformis*. Comparison of enzyme saturation constant, *K*_m(app)_, across treatments in strains **C.** *N. europaea* and **D.** *N. multiformis*. Data are presented as box plots, and different letters on top of each box plot denote significant differences (*P* < 0.05) in enzyme kinetic parameters within each subplot when tested using ANOVA, followed by posthoc analysis.


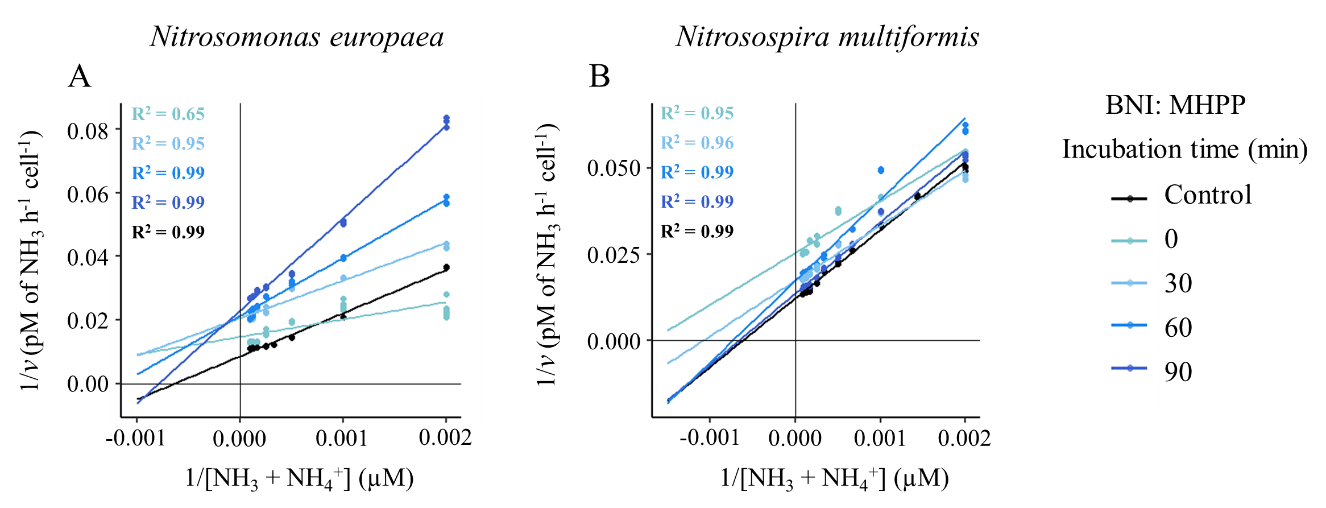


**Figure S7: Lineweaver-Burk plots depicting the influence of pre-incubation time with MHPP on enzyme kinetics in two species of ammonia-oxidising bacteria.** Lineweaver-Burk plots showing ammonium consumption rates after pre-incubation (0, 30, 60, and 90 min) with 3-(4-hydroxyphenyl)propionate (MHPP) in **A.** *N. europaea* and **B.** *N. multiformis.* Data are shown as dot and line plots.

**List of Supplementary Tables**

**Table S1. Stability of BNI compounds during enzyme kinetic assay.** BNI compound concentrations (µM, mean ± SD) of methyl 3-(4-hydroxyphenyl)propionate (MHPP), gallic acid (GA), and 6-methoxybenzoxazolinone (MBOA) were measured after 1 h incubation under abiotic (filter-sterilised medium without cells) and biotic (AOB cell suspensions) conditions, using LC-MS/MS analysis, to assess compound degradation during enzyme kinetic assays. Compound recovery rates were calculated to assess the detection ability. Significant statistical differences (*P* < 0.05) in compound concentrations were determined using two-way ANOVA (two-factorial, BNI and source effect).

| BNI compound | Source | Compound recovery (%) | Detected concentration using LC-MS/MS (µM) | Statistical significance |
| --- | --- | --- | --- | --- |
| MHPP | Abiotic | 136 | 271 ± 2 | a |
| MHPP | Biotic | 134 | 268 ± 3 | a |
| GA | Abiotic | 108 | 269 ± 4 | b |
| GA | Biotic | 102 | 256 ± 5 | b |
| MBOA | Abiotic | 85 | 21 ± 0 | c |
| MBOA | Biotic | 85 | 21 ± 1 | c |

**Table S2. Kinetic parameters for O₂ consumption in the AOB strains without BNI supplementation.** The table summarises the apparent Michaelis-Menten constant (*K*_m(app)_), representing the substrate concentration at which half-maximal velocity is achieved, and maximum velocities (*V*_max(app)_), representing the maximum rate of reaction, modelled for O₂ consumption in *N. europaea* and *N. multiformis* strains. Values are presented as mean ± standard deviation (n = 3). Different letters denote significant differences (*P* < 0.05) in enzyme kinetic parameters within measurement type when tested using ANOVA, followed by post hoc analysis.

| **Strain** | ***K*m(app) (µM)** | ***V*max(app) (pM h⁻¹ cell⁻¹)** |
| --- | --- | --- |
| *N. europaea* | 1108.2 ± 18.5 a | 104.5 ± 0.8 a |
| *N. multiformis* | 1878.9 ± 6.9 b | 87.3 ± 0.7 b |
|  |  |  |

**Table S3. Linearity of O₂ consumption rates.** The R² values for regression in the decrease of O₂ concentrations in cultures *N. europaea* and *N. multiformis*, exposed to 200 µM of 3-(4-hydroxyphenyl)propionate (MHPP) and 35 µM of 6-methoxy-2-benzoxazolinone (MBOA) at different ammonium concentrations and pre-incubation times (0, 30, 60, or 90 mins). Values are presented as means ± standard deviations (n = 3).

| Ammonium (µM) | Pre-incubation time (min) | MHPP (200 µM) | | MBOA (35 µM) | |
| --- | --- | --- | --- | --- | --- |
|  |  | *N. europaea* | *N. multiformis* | *N. europaea* | *N. multiformis* |
|  |  | R^2^ | R^2^ | R^2^ | R^2^ |
| 12000 | 0 |  | 0.985 ± 0.003 | 0.999 ± 0.004 | 0.999 ± 0.001 |
| 10000 | 0 | 0.978 ± 0.001 | 0.978 ± 0.001 | 0.998 ± 0.005 | 0.999 ± 0.003 |
| 8000 | 0 | 0.972 ± 0.005 | 0.972 ± 0.005 | 0.997 ± 0.002 | 0.998 ± 0.005 |
| 6000 | 0 | 0.964 ± 0.003 | 0.964 ± 0.003 | 0.998 ± 0.008 | 0.998 ± 0.009 |
| 4000 | 0 | 0.970 ± 0.007 | 0.970 ± 0.007 | 0.996 ± 0.001 | 0.995 ± 0.008 |
| 2000 | 0 | 0.960 ± 0.009 | 0.960 ± 0.009 | 0.993 ± 0.008 | 0.997 ± 0.013 |
| 1000 | 0 | 0.961 ± 0.010 | 0.961 ± 0.010 | 0.989 ± 0.02 | 0.995 ± 0.011 |
| 500 | 0 | 0.959 ± 0.015 | 0.959 ± 0.017 | 0.989 ± 0.023 | 0.987 ± 0.015 |
| 12000 | 30 |  | 0.982 ± 0.001 | 0.999 ± 0.001 | 0.999 ± 0.001 |
| 10000 | 30 | 0.993 ± 0.012 | 0.981 ± 0.005 | 0.999 ± 0.001 | 0.999 ± 0.001 |
| 8000 | 30 | 0.988 ± 0.031 | 0.985 ± 0.011 | 0.999 ± 0.001 | 0.999 ± 0.001 |
| 6000 | 30 | 0.984 ± 0.007 | 0.991 ± 0.013 | 0.999 ± 0.001 | 0.999 ± 0.001 |
| 4000 | 30 | 0.976 ± 0.009 | 0.978 ± 0.008 | 0.999 ± 0.001 | 0.999 ± 0.001 |
| 2000 | 30 | 0.975 ± 0.016 | 0.972 ± 0.011 | 0.999 ± 0.001 | 0.999 ± 0.001 |
| 1000 | 30 | 0.967 ± 0.027 | 0.969 ± 0.017 | 0.999 ± 0.001 | 0.999 ± 0.001 |
| 500 | 30 | 0.978 ± 0.038 | 0.978 ± 0.026 | 0.999 ± 0.001 | 0.999 ± 0.001 |
| 12000 | 60 |  | 0.999 ± 0.001 | 1.000 ± 0.000 | 1.000 ± 0.000 |
| 10000 | 60 | 0.999 ± 0.001 | 0.999 ± 0.001 | 1.000 ± 0.000 | 1.000 ± 0.000 |
| 8000 | 60 | 0.999 ± 0.001 | 0.999 ± 0.001 | 1.000 ± 0.000 | 1.000 ± 0.000 |
| 6000 | 60 | 0.999 ± 0.001 | 0.999 ± 0.001 | 1.000 ± 0.000 | 1.000 ± 0.000 |
| 4000 | 60 | 0.999 ± 0.001 | 0.999 ± 0.001 | 1.000 ± 0.000 | 1.000 ± 0.000 |
| 2000 | 60 | 0.999 ± 0.001 | 0.999 ± 0.001 | 1.000 ± 0.000 | 1.000 ± 0.000 |
| 1000 | 60 | 0.999 ± 0.001 | 0.999 ± 0.001 | 1.000 ± 0.000 | 1.000 ± 0.000 |
| 500 | 60 | 0.999 ± 0.001 | 0.999 ± 0.001 | 1.000 ± 0.000 | 1.000 ± 0.000 |
| 12000 | 90 | 1.000 ± 0.000 | 1.000 ± 0.000 |  |  |
| 10000 | 90 | 1.000 ± 0.000 | 1.000 ± 0.000 |  |  |
| 8000 | 90 | 1.000 ± 0.000 | 1.000 ± 0.000 |  |  |
| 6000 | 90 | 1.000 ± 0.000 | 1.000 ± 0.000 |  |  |
| 4000 | 90 | 1.000 ± 0.000 | 1.000 ± 0.000 |  |  |
| 2000 | 90 | 1.000 ± 0.000 | 1.000 ± 0.000 |  |  |
| 1000 | 90 | 1.000 ± 0.000 | 1.000 ± 0.000 |  |  |
| 500 | 90 | 1.000 ± 0.000 | 1.000 ± 0.000 |  |  |

**Table S4.** **Multiple reaction monitoring (MRM) summary**. Summary of MRM parameters used for the quantification of gallic acid (GA), methyl 3-(4-hydroxyphenyl)propionate (MHPP), and 6-methoxy-2-benzoxazolinone (MBOA) by LC-MS/MS analysis. For each analyte, the optimised settings for collision energy (CE), declustering potential (DP), and collision cell exit potential (CXP) are provided, along with the corresponding electrospray ionisation (ESI) mode (positive or negative). Q1 mass and Q3 mass are the  m/z ratios from quadrupole 1 and quadrupole 3. These parameters were selected to maximise sensitivity and selectivity for each compound during analysis.

| Compounds | ESI | Q1 mass (m/z) | Q3 mass (m/z) | CE | DP | Dwell time (msec) | CXP |
| --- | --- | --- | --- | --- | --- | --- | --- |
| GA | negative | 169 | 125.00 | -20 | -20 | 80 | 0 |
|  |  |  | 79.00 | -30 | -32 | 80 | 0 |
| MBOA | negative | 163.9 | 120.897 | -28 | -45 | 50 | -11 |
|  |  |  | 148.867 | -20 | -45 | 75 | -13 |
| MHPP | positive | 181 | 106.955 | 17 | 13 | 100 | 8 |
